# Exploring coronial determination of intent for poisoning-related deaths in Australia, 2001–2013

**DOI:** 10.1186/s12889-017-4633-9

**Published:** 2017-08-01

**Authors:** Kate Churruca, Rebecca Mitchell

**Affiliations:** Australian Institute of Health Innovation, Macquarie University, Level 6, 75 Talavera Road, North Ryde, Sydney, NSW 2109 Australia

**Keywords:** Coroners systems, Suicide, Drug and alcohol policy, Medico-legal databases, Coding and classification

## Abstract

**Background:**

In countries like the United States and the United Kingdom, systematic variation in the classification of intent in pharmaceutical poisoning deaths have been identified between jurisdictions. This study aimed to explore whether the coronial determination of intent (unintentional, intentional, undetermined) for pharmaceutical-related poisoning deaths may have affected death rates over time and by jurisdiction in Australia.

**Methods:**

A retrospective examination of mortality records in the National Coronial Information System (NCIS) during 1 January 2001 to 31 December 2013 was conducted. The NCIS is a national internet-based data storage and retrieval system for deaths that were notified to a coroner. Pharmaceutical deaths due to unintentional, intentional or undetermined intent were identified using the NCIS classification. Proportions of the different intent classifications and the mortality rates by intent over time were compared between jurisdictions.

**Results:**

There were 17,895 pharmaceutical-related poisoning deaths in Australia between 2001 and 2013 that had closed cases in the NCIS. Proportions of deaths classified as unintentional (48.3–66.3%), intentional (24.7–35.9%) and undetermined (6.7–24.7%) varied significantly among Australian jurisdictions. There were significant increases in the rate of classification of unintentional poisoning for some states, and significant increases in intentional poisoning classification in Western Australia, and decreases in New South Wales and Victoria. There was no significant change in classification of undetermined intent.

**Conclusions:**

Significant variation in classifications of intent, both between state jurisdictions and over time, may be the result of regional differences in demographics and increases in prescription drug misuse. However, the inconsistent use of ‘undetermined’ intent between state jurisdictions suggests coroners may experience varying difficulty in retrospectively ruling on intent in the equivocal circumstances of pharmaceutical poisoning. The widespread use of psychological autopsy may assist coroners to classify intent, while the implementation of new classifications for pharmaceutical poisoning death may overcome some of the inherent difficulty in intent classification and improve the potential for injury surveillance irrespective of intent.

## Background

Intentional self-harm and suicide represent a significant public health problem worldwide [1]. In Australia, death due to intentional self-harm occurs at a rate of 10.1 per 100,000 population [2], which is comparable with the United States (US), New Zealand and Canada, but higher than the United Kingdom (UK) and a number of other European and Asian nations [3]. Indeed, such figures likely under enumerate the total number of self-harm deaths, because it is often difficult for coroners and medical examiners, who investigate injury deaths, to make an unequivocal decision of the individual’s intent, such as whether the death may have been intentional or unintentional if the person has not left a note, or there is no documented history of mental illness [4, 5]. In addition to the availability of information affecting determination of intent, a coroner’s suspicions of suicide may be outweighed by social pressures, such as the stigma of suicide, moral or religious concerns, and, in some cases (e.g., suicide within 13 months of policy commencement), the loss of life insurance benefits [6, 7]. These issues likely lead some instances of intentional self-harm deaths to be misclassified as ‘undetermined’ or ‘unintentional’ [7].

Deaths involving pharmaceuticals can be especially difficult for coroners to determine intent [8], because while death may not have necessarily been the intended outcome in circumstances of drug overdose, the ingestion of an intoxicating, often harm-causing, substance was also not unintentional [9]. Furthermore, use of some medicinal drugs is understood to inhibit an individual’s mental capacity to form an intent with regard to death [7]. Therefore, while substance use is a risk factor for suicide, the involvement of drugs in a death may reduce the likelihood that the death is classified as suicide [10]. Any instance of coroner misclassification of intent not only affects the estimation of suicide numbers, but also unintentional poisoning, making it difficult to assess each of their burdens. Misclassification of unintentional and intentional poisoning may impact upon the ability to understand risk factors for both, and gain support for, and adequately tailor, prevention initiatives [7], which have traditionally focused on different factors: environment for unintentional injuries and behaviour for intentional injury [11].

In the US, the frequency of classification of deaths due to ‘undetermined’ intent has been found to vary considerably by state (from <4% to 85% of poisoning deaths), as has the level of documentation of specific drugs on the death certificate, fluctuations that may be due to differing resources, investigatory systems (e.g., centralized medical examiner versus county coroner), methods of documenting, and context [12]. Breiding and Wiersema [13] also found that states with the highest rates of undetermined intent ranked behind most others on mean mortality rates of both intentional and unintentional pharmaceutical poisoning deaths. Furthermore, an association has been established between the decline in the suicide rate and the large increase in unintentional poisonings from 1987 to 2006, which suggests some potential suicides are being classified as unintentional deaths [14]. Comparable to these trends, in the UK, McLean [15] found that jurisdictions with the lowest rates of suicide had the highest rates of ‘narrative’ verdicts, which involves the factual reporting of circumstance for a death that does not simply fit standard verdicts (e.g., natural causes, suicide); on the other hand, jurisdictions with the lowest rates of narrative verdicts had the highest rates of suicide.

In Australia, while deaths due to unintentional pharmaceutical poisoning were on the decline to 2002, from 6.8 deaths per 100,000 population to 3.4 deaths, they have since been increasing (4.5 deaths per 100,000 in 2009–10), corresponding with increases in the use and misuse of prescription opioids [16, 17]. However, little is known about whether there are differences in the classification of intent in Australia for pharmaceutical poisoning, such as less frequent classification of intentional poisoning or more frequent use of undetermined intent for pharmaceutical poisoning. As with countries like the US and UK, there is a lack of standardisation of the procedures for determining intent, with Australian jurisdictions (i.e., state or territory coronial office) having different legislation and coroners not typically legally required to determine intent. Coders for the National Coronial Information System (NCIS), who categorize cases of coroner reported death nationally, have previously reported difficulties in coding intent, particularly for overdose deaths, and coders vary in the documentation and procedures they rely on for this classification [18]. This suggest the likelihood of variability between jurisdictions [19] impacting on injury surveillance of pharmaceutical poisoning by intent. This study aims to explore whether the coronial determination of intent for pharmaceutical-related poisoning deaths may have affected mortality incidence rates for different intent classifications over time and by jurisdiction in Australia.

## Method

A retrospective examination of mortality records in the NCIS during 1 January 2001 to 31 December 2013 was conducted. Ethical approval was obtained from the Macquarie University Human Research Ethics Committee (reference no: 5,201,500,660), the Victorian State Government Justice Human Research Ethics Committee (CF/15/16426) and the Western Australia Coronial Ethics Committee (EC16/2015).

### Data collection

The NCIS is a national internet-based data storage and retrieval system for deaths that were notified to a coroner. All deaths not presumed to have a natural cause (including injury-related deaths) should be notified to a coroner in Australia. The NCIS contains information on every death reported to an Australian coroner since July 2000 (January 2001 for Queensland). Within the NCIS, detailed information regarding the circumstances of the death is available on ‘closed cases’ (i.e., coronial cases finalized by a coroner) and minimal information is available on ‘open cases’ (excluding Western Australia, where information is only available on ‘closed cases’).

The NCIS includes information on the cause and circumstances of the death (e.g., mechanism of injury, intent), and demographic information of the person who died, with many the death records having reports (autopsy, toxicology, police) and coronial findings attached. Pharmaceutical-related poisoning was identified using the NCIS classifications for mechanism of injury of *poisoning by solid substance (NCIS: 6.01.1)* OR *poisoning by liquid substance* (NCIS: 6.01.2) AND *object or substance producing injury is pharmaceutical substance for human use* (NCIS: 20, excluding alcohol: 20.40) [20]. A search of *poisoning by multiple substances* (NCIS: 6.01.4) was also conducted; any cases containing a pharmaceutical in their *object or substance producing injury* were included. Intent was identified using the NCIS intent at case completion of *unintentional* (NCIS: 1), *intentional self-harm* (NCIS: 2), or *undetermined intent* (NCIS: 7). Any cases classified with intent as *unlikely to be known* (NCIS: 999), or with intent left blank, were also coded as undetermined intent [5]. All other possible intents (NCIS: 3–6) were classified as ‘other’.

### Data management and analysis

All analyses were performed using SAS version 9.4 [21]. Descriptive statistics were conducted. Chi-square analysis was conducted to examine the differences in the proportion of intent classifications by jurisdiction for the whole 13-year period. Crude mortality incidence rates over time were calculated using denominator data obtained from the ABS population estimates [22] for the whole of Australia, and for the individual states of New South Wales (NSW), Victoria (VIC), Queensland (QLD), South Australia (SA), and Western Australia (WA). Tasmania (TAS), the Northern Territory (NT) and the Australian Capital Territory (ACT) were excluded from rates due to low cell frequency. Due to over-dispersion, negative binomial regression analyses using the number of deaths as the dependent variable and year of death and age group as independent variables with an offset of the log of the population were used to examine the statistical significance of changes in the trend over time in mortality incidence rates by intent for the whole of Australia and then within each state [23]. Age-standardised mortality rates were not able to be calculated due to <5 cell size by age group and gender for some jurisdictions.

## Results

There were 17,895 deaths as a result of pharmaceutical poisoning in Australia from 2001 to 2013 identified using the NCIS. The number of deaths due to pharmaceutical poisoning increased over this 13-year period, peaking in 2012 with 1712 deaths. Close to two-thirds of the sample were male (64.3%). The highest proportion of deaths occurred in those in the middle adulthood years of 30–49 years (50.4%). Unintentional poisoning was the most commonly classified intent, with 9951 deaths (55.6%), and NSW had the highest number of pharmaceutical poisoning deaths (Table 1).

**Table 1:** Characteristics of individuals who died as a result of pharmaceutical poisoning, Australia, 2001–2013

	All persons (17,895)		Male (11,509)		Female (6386)	
	n	%	n	%	n	%
Year of death						
2001	1085	6.1	678	5.9	407	6.4
2002	1086	6.1	700	6.1	386	6.0
2003	1169	6.5	750	6.5	419	6.6
2004	1254	7.0	837	7.3	417	6.5
2005	1355	7.6	881	7.7	474	7.4
2006	1196	6.8	755	6.6	441	6.9
2007	1435	8.0	871	7.6	564	8.8
2008	1520	8.5	1002	8.7	518	8.1
2009	1602	9.0	1048	9.1	554	8.7
2010	1465	8.2	945	8.2	520	8.1
2011	1549	8.7	1022	8.9	527	8.3
2012	1712	9.6	1099	9.6	613	9.6
2013	1467	8.2	921	8.0	546	8.6
Age groups^a^						
0–19	453	2.5	300	2.6	153	2.4
20–29	3227	18.0	2398	20.8	829	13.0
30–39	4766	26.6	3409	29.6	1357	21.3
40–49	4267	23.8	2771	24.1	1496	23.4
50–59	2861	16.0	1559	13.6	1302	20.4
60–69	1230	6.9	593	5.2	637	10.0
70+	1088	6.1	476	4.1	612	9.6
Intent						
Unintentional	9951	55.6	6974	60.6	2977	46.6
Intentional	5331	29.8	2966	25.8	2365	37.0
Undetermined	2534	14.2	1534	13.3	1000	15.7
Other^b^	79	0.4	35	0.3	44	0.7
State						
NSW	5264	29.4	3404	29.6	1860	29.1
VIC	4411	24.7	2854	24.8	1557	24.4
QLD	3289	18.9	2080	18.1	1209	18.9
SA	1374	7.7	830	7.2	544	8.5
WA	2486	13.9	1685	14.6	801	12.5
TAS	586	3.3	341	3.0	245	3.8
NT	216	1.2	161	1.4	55	0.9
ACT	269	1.5	154	1.3	115	1.8

^a^Three male cases are not reported because they were missing age details

^b^Other intent includes Legal Intervention, Operations of War, Civil Conflict and Acts of Terrorism, Other Specified Intent [20]

**Fig. 1:**
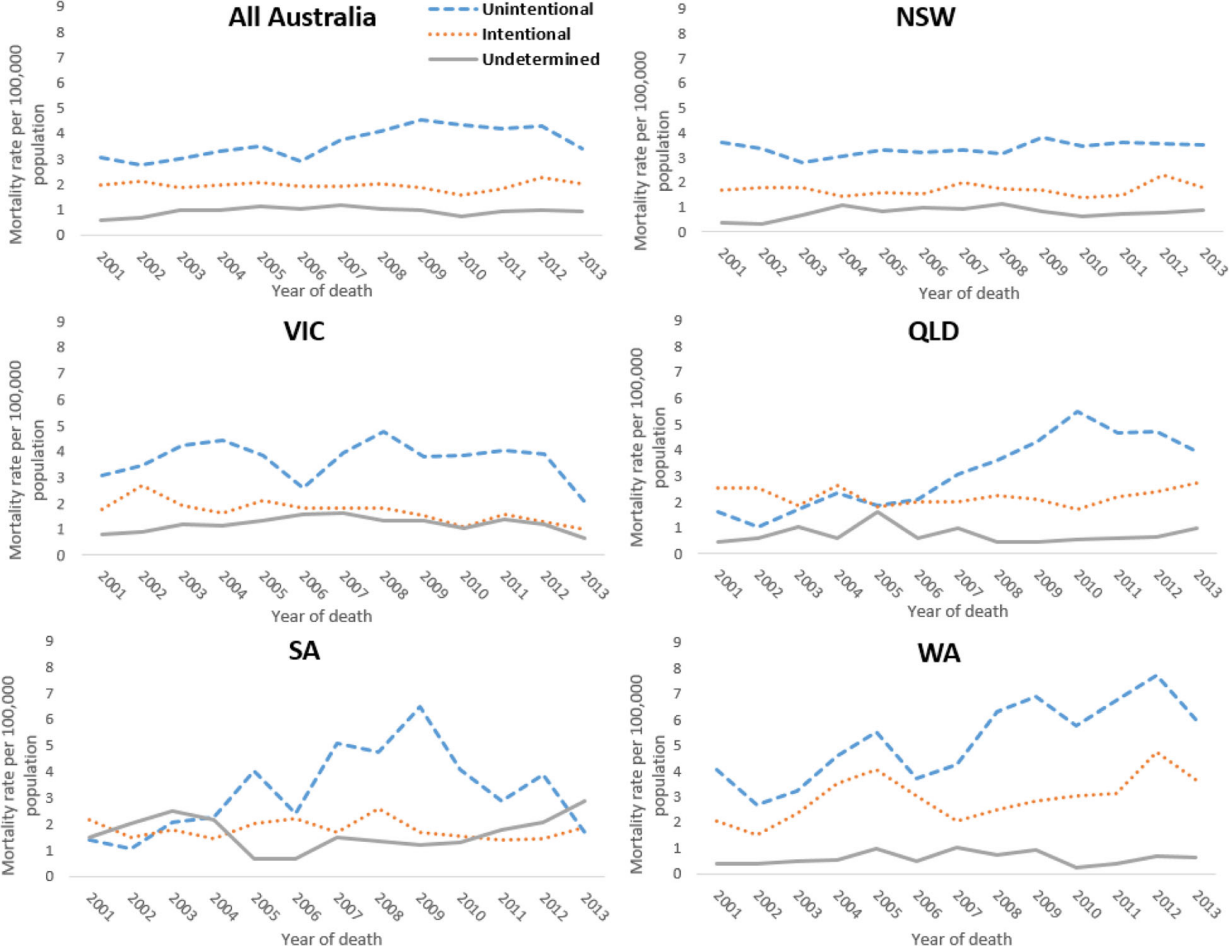
Crude rates for deaths due to pharmaceutical poisoning by intent in Australia and by state for period 2001–2013

Examination of the frequency of intent classification by state suggested some variability. The proportion of deaths that were classified as unintentional (48.3–66.3%), intentional (24.7–35.9%) and undetermined (6.7–24.7%) varied significantly for the eight Australian jurisdictions during the 13-year study period of study (Table 2). Crude mortality rates for unintentional, intentional, and undetermined intent further indicated variability in the classification of intent over time and between the different states of Australia (Fig. 1). Most of this variation was in the classification of unintentional poisoning. This variation in unintentional poisoning was evident for all Australia, as well as between Australian states (e.g., variation between WA and VIC) and within most states over time, with NSW the only exception to this. Results of the individual negative binomial regression analyses showed significant increases in the rate of classification of unintentional poisoning for the model with the whole of Australia, and then models for a number of states (QLD, SA, WA). The rate of intentional pharmaceutical poisoning also significantly increased for the model of the whole of Australia and for that of WA; on the other hand, classification of intentional poisoning significantly decreased in NSW and VIC. While changes in the rates of undetermined intent varied between the models for individual states, with some rates increasing (NSW, VIC, WA) and others decreasing (QLD, SA), none of these changes were significant (Table 3).

**Table 2:** Frequency and percentage of deaths through pharmaceutical poisoning by intent for all Australia and each state or territory, 2001–2013

Location	Intent Classification^a^						χ^2^ (df)
	Unintentional		Intentional		Undetermined		
	n	%	n	%	n	%	
All Australia	9951	55.9	5331	29.9	2534	14.2	
States							
NSW	3020	57.6	1529	29.2	693	13.2	404.60 (14)****
VIC	2473	56.4	1116	25.5	793	18.1	
QLD	1718	52.4	1178	35.9	386	11.8	
SA	664	48.5	366	26.8	338	24.7	
WA	1479	59.7	835	33.7	165	6.7	
TAS	299	51.6	179	30.7	102	17.6	
NT	121	56.0	62	28.7	33	15.3	
ACT	177	66.3	66	24.7	24	9.0	

****p < 0.0001

^a^‘Other’ intent not reported

**Table 3:** Regression of rates of change in intent classification for pharmaceutical poisoning deaths in Australia and by state, 2001–2013

Location	Change	95% CI
All Australia		
Unintentional	3.47****	2.49–4.46
Intentional	1.83**	0.76–2.91
Undetermined	0.22	−0.96 - 1.41
NSW		
Unintentional	0.99	−0.70 - 2.71
Intentional	−5.18**	−8.72 - -1.50
Undetermined	1.43	−0.91 – 3.82
VIC		
Unintentional	−0.07	−1.99 - 1.88
Intentional	−5.05****	−7.30 - -2.75
Undetermined	0.98	−1.02 – 3.03
QLD		
Unintentional	10.26****	7.91–12.65
Intentional	0.84	−1.32 – 3.05
Undetermined	−0.85	−3.59 – 1.98
SA		
Unintentional	3.95***	1.64–6.31
Intentional	1.37	−0.99 - 3.79
Undetermined	−0.49	−3.21 – 2.32
WA		
Unintentional	6.66****	3.90–9.50
Intentional	4.40***	2.14 - 6.72
Undetermined	0.61	−3.60 – 4.99

****p < 0.0001, ***p < 0.001, **p < 0.01

## Discussion

This study explored whether the coronial determination of intent for pharmaceutical-related poisoning deaths may have affected mortality incidence rates over time and by state in Australia using the NCIS data collection from 2001 to 2013. There was a rise in the number of pharmaceutical poisoning deaths over the 13-year period of study, with an examination of the annual percent change in mortality rates for both unintentional and intentional poisoning also showing significant increases over time, except for intentional poisoning in NSW and VIC where there were significant decreases. Overall, the number of pharmaceutical deaths identified in this study were similar to those found by Henley and Harrison [17], who reported 1496 cases of pharmaceutical poisoning mortality for 2009–10 and comparable proportions of unintentional, intentional and undetermined intent deaths.

The classification of intent, when collapsed across the 13-year period of study, was found to vary significantly between Australian states, with much higher proportions of unintentional poisoning in the ACT, and undetermined intent in VIC, SA, and TAS. It is unclear at this stage to what extent some of this state-based variation in the intent classification for poisoning deaths reflects real difference in manner of death between different states versus inconsistent classification practices between jurisdictions. There is considerable variation in the population characteristics among Australian states and territories. For example, the ACT has higher number of females and younger people in their population, and approximately 34% are employed by the Commonwealth Government of Australia [24]. On the other hand, in WA and the NT, a larger proportion of the population is male, and, for WA in particular, employed in high-paying, ‘fly-in, fly-out’ mining work [25, 26]. These regional differences have likely affected the type of drug and patterns of drug-taking [27], hence contributing to the different proportions of intent classifications between states (e.g., higher proportion of unintentional poisoning in ACT, and intentional in WA). At the same time, the variation between some states in the proportion of undetermined intent classification (i.e., 6.7–24.7%) seems to suggest more than just an increased frequency of ambiguous deaths within these jurisdictions. In this vein, a US study by Warner, et al. [12] noted that large differences between states in the use of the undetermined intent classification were likely due to a lack of standardisation in the methods of investigating deaths and the criteria used to determine intent.

The present study also found changes in the classification of intent for pharmaceutical deaths for some states over time, such as increases in both intentional and unintentional poisoning in WA, and decreases in intentional poisoning in VIC and NSW. However, contrary to trends from the US reported by Breiding and Wiersema [13] and Rockett, et al. [14], significant declines in the rate of intentional poisoning, such as in NSW and VIC, did not correspond with increases in either rates of unintentional or undetermined intent. Indeed, changes in the rate of classification of undetermined intent for all states were nonsignificant, though graphically crude rates for SA and QLD did show the anticipated trend of a negative relationship between undetermined intent and intentional poisoning. Finally, for WA rates of both unintentional and intentional poisoning rose over time, but did not correspond to any significant changes in the rate of undetermined intent.

Overall, findings suggest mortality incidence rates may be affected by some variations in the classification of intent in pharmaceutical poisoning deaths between coronial jurisdictions in Australia, specifically regarding the frequency with which the classification of undetermined intent is utilized between some states. The evidence to suggest changes in classification of intent over time is more tentative, and does not follow the trend found in the US [14]; instead there were significant increases in both unintentional and intentional poisoning. The trends identified in this study may therefore reflect real changes in the incidence of pharmaceutical poisoning for the different intent types. For example, the increase in unintentional poisoning over time may reflect changes in manner of death between states due to differences in their sociodemographic characteristics, and particularly increases in the misuse of prescription pharmaceuticals, which are often classified under ‘unintentional’ [28]. Future research exploring differences in patterns of drug-taking and pharmaceutical poisoning between Australian states over time may provide further insights into the reasons for diverging trends among jurisdictions with regard to intent.

Nevertheless, the large variability in usage of the classification of ‘undetermined intent’ in this study between coronial jurisdictions suggests there may be a lack of consensus among coroners in Australian states about how to classify some deaths resulting from pharmaceutical poisoning. This may still limit the ability to accurately determine the extent of intentional and unintentional poisoning at a state and national level, and to then address them accordingly through the identification of risk factors and targeted interventions [6, 7]. The more widespread use of psychological autopsies might be beneficial in reducing the use of ‘undetermined’ intent at the time in which coroners make their determination, by providing them more information and expertise to make an accurate decision [4]. Psychological autopsy involves comprehensive psychological information about the deceased person being collected through extensive interviews with family members and other close intimate persons, as well as through medical, psychiatric and legal records; this information may be assessed by psychiatrists and other mental health professionals to accurately determine the role of intent in the death [29]. While not currently standard practice in nations like Australia [30], they have been found to improve the precision of classification of intent. Moreover, psychiatrists and suicide researchers have been found more likely to identify deaths that were previously classified by coroners as undetermined intent to be intentional [31]. However, the implementation of psychological autopsies would require the allocation of greater resources to coronial offices. As a starting point, national coronial practice guidelines and aids regarding the classification of intent [6], particularly for pharmaceutical poisonings, may be useful in improving consistency among the classifications between jurisdictions. The linkage of administrative health datasets may also provide an opportunity into the future to gather additional information to support the determination of intent, such as the deceased having a record of hospitalisation for self-harm, drug use, or mental illness.

As an alternative to standardisation of existing procedures, the implementation of new concepts to classify deaths due to pharmaceutical poisoning death may overcome some of the inherent difficulty in attempting to retrospectively determine the role of intent in equivocal circumstances. In this vein, Rockett, et al. [14] have proposed the addition of a category, *death from drug self-intoxication* (DDSI), which would apply across intent classifications to indicate deaths where pharmaceuticals were deliberately ingested for purposes of intoxication (e.g., overdose deaths). Similar to this, using examples of equivocal gunshot death, Obenson [32] suggested that the classification of suicide could be supplanted by that of ‘self-inflicted’ death, which would carry less stigma, reduce the subjectivity of medical examiners and pathologists ruling on intent, and defer this decision to other more qualified or better resourced facilities (e.g., departments conducting psychological autopsies).

Hence, the use of a DDSI style classification in Australia would allow for better tracking of the problematic use of pharmaceuticals and sidestep some of the difficulty for coroners in determining a construct like intent in pharmaceutical poisoning, where death might not be intended but intoxication is, and the potential for self-harm is recognized. This categorisation could also trigger resourcing for further investigation of DDSI deaths to determine intent, such as through psychological autopsy. Monitoring pharmaceutical usage has received increasing attention in Australia recently [33], with the rise of prescription drug misuse. The implementation of DDSI classification could work alongside other proposed strategies such as real-time prescription drug monitoring, which attempts to track and subsequently address problematic prescribing, dispensing and use of some prescription drugs to avoid unintentional poisoning [33].

These findings were strengthened by the population-based nature of the study, the use of a large cohort and inclusion of data from a relatively long period of time, which thereby enabled greater capacity to identify trends. At the same time, there were some limitations to this study. First, due to the small numbers of deaths for some age groups by sex, age and sex standardized mortality rates were not able to be calculated for each intent classification, which may have affected comparisons between some states. The ICD-10 codes, which are used by the ABS to classify mortality data for official reporting, were also not used in this study to select cases of pharmaceutical poisoning nor to classify intent. This may lead to some discrepancies between the number and rate of poisoning deaths identified in the current study and other reported Australian national mortality figures using Cause of Death-Unit Record File mortality data, particularly as there is the possibility of incorrect classification of poisoning deaths in the NCIS. However, the ABS relies upon coronial investigations and NCIS data for their classification of cause of death and intent [5]. Moreover, the similarity in the number of poisoning deaths and proportion of different intent classifications in the current study to those identified by Henley and Harrison [17] suggests any such discrepancies were minor.

This study was confined to the examination of NCIS closed cases, which may have reduced the number of pharmaceutical poisoning-related deaths, especially for later years where coronial investigations of deaths may not yet have concluded [34]. This may have affected rates of intent classification over time and particularly differences between states. For example, while Studdert, et al. [35] found the average length of time for closure of coronial cases involving suicide or undetermined intent was relatively short, case closure was affected by jurisdiction; however, this was only for the smaller subset of cases requiring a full inquest. It is also possible that the numbers of deaths by intent reported in this study reflect not only cases of intent as classified by coroners, but NCIS coders too, with De Leo, et al. [6] finding that in 29% of the 988 NCIS cases they reviewed, the coroner made no reference to intent. However, this issue is reportedly more likely to occur for deaths with less equivocal circumstances (e.g. hanging, motor vehicle exhaust) than pharmaceutical poisoning deaths. Finally, in this study 5.0% of deaths had no classification for their *intent at case completion*. It is unclear whether this was due to oversight, reluctance among some NCIS coders to classify intent, or some other reason, and following the ABS guidelines, these blank cases were treated as undetermined intent [5]. However, it is worth noting that the proportion of deaths that did not have a classified intent at case completion varied by jurisdiction (2.3–11.1%), which may have affected rates of undetermined intent by state.

Finally, research suggests that the way in which intent is classified over time is associated with the type of drugs ingested (e.g., prescription or recreational) [13]. Any associations between intent classification and the types of pharmaceuticals involved in the death were unable to be examined for the present study, however, because 35.8% of the sample had their *primary pharmaceutical object or substance producing injury* classified as *other specified multiple substances* (NCIS: 20.50.98), which precluded meaningful analysis of any relationship between specific drugs and intent classification. This is also unfortunate because the ability to identify associations between specific and multiple pharmaceuticals and intent may have significant implications for prevention of injury and death [12]. This issue resonates with previous research showing that even on coroner reports and findings, drugs and specific drugs are often underreported as a cause of death and, hence, underacknowledged as an opportunity for future prevention [12, 36]. However, usage of multiple drugs is common practice, particularly among older people in Australia [37], with around two-thirds of individuals aged 60 years or older using four or more medications [38]. As such, revising coding practices for pharmaceutical deaths documented in medico-legal databases, such as the NCIS, to allow for the coding of individual drugs in circumstances were multiple drugs are involved in the death is worthwhile pursuit to enable further research on the effect of drug type on intent classification, in addition to future injury surveillance and prevention. Alternatively, the use of Cause of Death-Unit Record File data, which involves classification by the ABS using ICD-10 codes, would enable future research to explore whether drug-type affects the way coroners determine intent.

## Conclusion

This study found that there were significant differences between jurisdictions in the proportion of pharmaceutical-related poisoning deaths classified as intentional, unintentional and undetermined intent during 2001–2013 in Australia. Moreover, there were significant changes in the classification of intent for some states over time, with decreases in intentional poisoning (VIC, NSW), and, particularly, increases in unintentional poisoning overall in Australia and some states (QLD). The variability between jurisdictions in the use of the classification of ‘undetermined intent’ suggests there may be a need for greater standardisation of procedures for classifying deaths due to pharmaceutical poisoning in Australia.

## Abbreviations

ABS: Australian Bureau of Statistics
ACT: Australian Capital Territory
ICD-10: International Statistical Classification of Diseases and Related Health Problems version 10
NCIS: National Coronial Information System
NSW: New South Wales
NT: Northern Territory
QLD: Queensland
SA: South Australia
TAS: Tasmania
VIC: Victoria
